# Supplementary material for: Comprehensive occupational health services for healthcare workers in Zimbabwe during the SARS-CoV-2 pandemic
Source: PLoS One. 2021 Nov 23;16(11):e0260261. doi: 10.1371/journal.pone.0260261 (PMC8610265; doi:10.1371/journal.pone.0260261)
Supplement: S1 Table — (DOCX) [file pone.0260261.s001.docx]

Supplementary table 1

*Demographic and clinical characteristics of participants not tested, testing negative, and testing positive for, SARS-CoV-2*

| Variable | SARS-CoV-2 test  not done | SARS-CoV-2 test  positive | SARS-CoV-2 test negative |
| --- | --- | --- | --- |
| Total | 741 (77.9%) | 12 (1.3%) | 198 (20.8%) |
| Male | 125 (16.9%) | 3 (25.0%) | 39 (19.7%) |
| Clinical occupation | 408 (55.1%) | 10 (83.3%) | 127 (64.1%) |
| HIV positive | 54 (7.3%) | 2 (16.7%) | 16 (8.1%) |
| Hypertension | 294 (39.7%) | 6 (50.0%) | 82(41.6%) |
| HbA1c >6.5 or diabetes | 82 (11.1%) | 0 (0.0%) | 16 (8.1%) |
| BMI > 25 | 458 (62.0%) | 9 (75.0%) | 115 (58.4%) |
| COVID contact | 32 (4.3%) | 10 (83.3%) | 80 (40.4%) |
| If yes, appropriate PPE? | 24/32 (75.0%) | 8/10 (80.0%) | 38/80 (47.5%) |
| Dry cough | 4 (0.5%) | 2 (16.7%) | 43 (21.7%) |
| Productive cough | 0 (0.0%) | 0 (0.0%) | 15 (7.6%) |
| Cough 2+ weeks | 0 (0.0%) | 0 (0.0%) | 3 (1.5%) |
| TB contact | 0 (0.0%) | 0 (0.0%) | 6 (3.0%) |
| Fever | 0 (0.0%) | 1 (8.3%) | 37 (18.7%) |
| Weight loss | 7 (0.9%) | 0 (0.0%) | 13 (6.6%) |
| Night sweats | 3 (0.4%) | 0 (0.0%) | 16 (8.1%) |
| Loss of taste | 1 (0.1%) | 1 (8.3%) | 39 (19.7%) |
| Loss of smell | 0 (0.0%) | 2 (16.7%) | 21 (10.6%) |
| Fatigue | 4 (0.5%) | 3 (25.0%) | 64 (32.3%) |
| Sneeze | 2 (0.3%) | 6 (50.0%) | 70 (35.4%) |
| Runny nose | 4 (0.5%) | 3 (25.0%) | 37 (18.7%) |
| Headache | 11 (1.5%) | 2 (16.7%) | 98 (49.5%) |
| Joint pains | 3 (0.4%) | 2 (16.7%) | 48 (24.2%) |
| Sore throat | 5 (0.7%) | 0 (0.0%) | 67 (33.8%) |
| Diarrhoea | 0 (0.0%) | 3 (25.0%) | 12 (6.1%) |
| Mouth ulcers | 0 (0.0%) | 1 (8.3%) | 6 (3.0%) |
| Swollen glands | 0 (0.0%) | 0 (0.0%) | 2 (1.0%) |
| Any COVID symptom | 25 (3.4%) | 8 (66.7%) | 177 (89.4%) |
| Fever >37.5 | 1 (0.1%) | 0 (0.0%) | 9 (4.5%) |
| Knowledge score (median, IQR) | 8 (7-9) | 10 (9-10) | 9 (8-10) |
