# Supplementary material for: Comprehensive occupational health services for healthcare workers in Zimbabwe during the SARS-CoV-2 pandemic
Source: PLoS One. 2021 Nov 23;16(11):e0260261. doi: 10.1371/journal.pone.0260261 (PMC8610265; doi:10.1371/journal.pone.0260261)
Supplement: S2 File — (PDF) [file pone.0260261.s003.pdf]

**Q00**	Consent	Has verbal consent been obtained?<br>*If verbal consent declined , then do not proceed with the questionnaire*	Yes ☐ No ☐
**Q0a**	ID	ID	IC☐☐☐☐
**Q01**	DATE	Date form completed (dd/mm/yyyy)	☐☐/☐☐☐/20☐☐
**Q02**	SEX	Sex	Male☐ Female☐
**Q03**	AGE	Age, years, at last birthday	☐☐
**Q04**	LOC	Work location	Poly clinic ☐<br>Hospital ☐<br>Community setting ☐<br>Other ☐<br>Please specify
**Q05**	EM	Employer	City health service☐<br>Government health services (excluding city health) ☐<br>Private health facility (hospitals or clinics) ☐<br>NGO☐<br>Other☐
**Q05**	CLIN	Clinic /Hospital (we will add to the list)	Budiriro☐<br>Rujeko☐<br>Rutsanana☐<br>Mufakose ☐<br>Kuwadzana ☐<br>Warren Park ☐<br>Mabvuku ☐<br>Hatcliffe ☐

			Other ☐<br>Specify Other____________________
**Q06**	Occ	What is your role at the health facility	Nurse ☐<br>Midwife ☐<br>Community health care worker ☐<br>Security ☐<br>Administration/Clerk/IT ☐<br>Nurse Aide ☐<br>Laboratory Tech ☐<br>Radiographer ☐<br>Doctor ☐<br>Cleaner ☐<br>Porter ☐<br>Service/maintenance team ☐<br>Student nurse/midwife ☐<br>Student doctor ☐<br>Police/army ☐<br>Other ☐<br>Specify-----------------------------
**Q6a**	Duration	How long have you been working in your current job/role?	_____Years<br>_____Months
**Q07**	EDULEV	What is the highest level of education you have achieved	Did not complete primary/no school ☐<br>Primary ☐<br>Secondary 0 level ☐<br>Secondary A level ☐<br>Diploma after secondary ☐<br>University ☐
**Clinical**			
**Q08**	MEDICAL	Do you have any known medical conditions?	Yes ☐ No ☐
**Q8A**	Medical contd	If yes, please tick all that applies	Diabetes ☐

			Hypertension ☐<br>Epilepsy ☐<br>HIV ☐<br>Cardiovascular disease/past stroke ☐<br>Renal disease ☐<br>Asthma/COPD ☐<br>**Past** TB ☐<br>Past or present malignancy ☐<br>Don't wont to answer ☐<br>Other ☐<br>Specify-----------------------------
**Q8B**	Med	What medication do you take regularly (excluding short course antibiotics and Cotrim)	ART ☐<br>Oral antidiabetics ☐<br>Insulin ☐<br>Antihypertensives ☐<br>Heart medication (excluding antihypertensives) ☐<br>Oral steroids ☐<br>Inhalers ☐<br>Antiepileptic meds ☐<br>Oral contraceptive/implant ☐<br>Treatment for TB ☐<br>INH prophylaxis ☐

			Other ☐<br>Specify------------------------------
**Q09**	CIG	Do you smoke cigarettes? If yes, how many per day	Yes ☐ No ☐<br>___________per day
**HIV**			
**Q11**	PREVHIV	Have you ever had an HIV test	Don't want to answer ☐ Yes ☐ No ☐
**Q11a**	PREVHIV	If yes, what was the result of the last test	Pos☐<br>Neg☐<br>Don't want to answer☐
**Q11b**	ART	If positive, are you taking ART	Don't want to answer ☐ Yes ☐ No ☐
**Q11c**	DURART	How long have you been taking ART (year)	___________years
**Q11d**	VL	When did you have last your viral load checked (months)	___________years<br>Never☐<br>Don't want to answer☐
**Symptoms of respiratory infection/TB , COVID-19 risk factors**			
**Q13**	RESP	Do you have any of the following symptoms? (Tick all that apply)<br>(Generate WHO TB Symptom screen from here)	Cough (dry) ☐<br>Cough (productive) ☐<br>Cough > 2 weeks ☐<br>Contact of TB case☐<br>Fever ☐<br>Weight loss☐<br>Night sweats☐

			Loss of taste☐<br>Loss of smell☐<br>Fatigue ☐<br>Sneezing☐<br>Runny nose☐<br>Headache☐<br>Painful joints/muscles☐<br>Sore throat☐<br>Diarrhoea☐<br>Mouth ulcers☐<br>Swelling of lymph glands ☐<br>Contact of TB case☐
**Q14**	COV CON	Have you to your knowledge treated a patient with COVID-19 or come into contact with somebody who may have COVID-19 in the past 2 weeks	Yes☐<br>No☐
**Medical Aid**			
**Q15**	Med Aid	Do you have medical aid?	yes☐<br>No☐
**Q16**	MedAid type	CIMAS<br>PSMAS<br>First Mutual<br>Alliance Health<br>BONVIE<br>OTHER	Yes ☐ No ☐<br>Yes ☐No☐<br>Yes ☐ No ☐<br>Yes ☐No☐<br>Yes ☐No☐<br>Specify-------------

Clinical examinations			
**Q17**	O2	Sats (%)	☐☐☐
**Q18**	Temp	Temp (©)	☐☐,☐
**Q19**	Weight	Weight(kg)	☐☐☐,☐
**Q20**	Height	Height(cm)	☐☐☐
**Q21**	sBP	BP (mmHg) - systolic	☐☐☐
**Q22**	dBP	BP (mmHg) - diastolic	☐☐☐
**Q23**	HbA1c	HbA1c %	☐☐,☐
**Q24**	HIV_test	HIV test	Blood-based test by nurse ☐<br>OMT by client on site ☐<br>OMT provided to client for off-site testing ☐<br>HIV testing not done ☐
**Q25**	HIV_results	HIV test result	Pos ☐<br>Neg ☐<br>Not applicable ☐
**Q26**	SSQ	SSQ-score	______
**Q27**	COVID_s	COVID19_Symptoms	Yes ☐<br>No ☐
**Q28**	SWAB	Specimen for SARS-CoV-2 testing taken	Yes ☐<br>No ☐

**Q29**	WHO_TB	WHO TB symptom screen positive?	Yes☐<br>No☐
**Q30**	Sputum	Sputum samples obtained	Yes☐<br>No☐
**Q31**	Referral	Referred to	Chronic disease (HTN)☐<br>DM☐<br>HIV☐<br>Friendship bench☐<br>Other☐<br>Specify_____

**Q00**	Consent	Has verbal consent been obtained?<br>*If verbal consent declined , then do not proceed with the questionnaire*	Yes ☐ No ☐
**Q0a**	ID	ID	IC☐☐☐☐
**Q01**	DATE	Date form completed (dd/mm/yyyy)	☐☐/☐☐☐/20☐☐
**Use of PPE in health facility**			
**Q2**	Masks	Have you been given a mask to use at your place of work?<br>If yes, please tick which type	Yes ☐ No ☐<br>Surgical mask☐<br>FFP3 (N95) Mask☐<br>KN95 Mask☐<br>Cloth mask☐
**Q3**	Masks continued	If yes, are you given a new one every day? (if not a cloth mask)	☐ Yes ☐ No
**Q4**	Masks Avail	Has there been a time when there are no masks available?	☐ Yes ☐ No
**Q5**	Water	Is there running water from the tap every day at your place of work?	Yes, all the time ☐<br>Yes, intermittently☐<br>Almost never☐<br>No☐
**Q6**	Soap	Is there hand soap available every day at your place of work?	Yes, all the time ☐<br>Yes, intermittently☐<br>Almost never☐<br>No☐
**Q7**	Sanitizer	Is there hand sanitiser at your place of work?	Yes, all the time ☐<br>Yes, intermittently☐<br>Almost never☐<br>No☐

**Q8**	Bleach	Does your place of work have bleach to clean down surfaces throughout the day?	Yes, all the time ☐<br>Yes, intermittently ☐<br>Almost never ☐<br>No ☐
**Q9**	Training	Have you received training infection and prevention control procedures since COVID-19 pandemic began?	Yes ☐ No ☐
**Household members (to be asked for those who require COVID testing)**			
**Q10**	Household members	How many people have been living in your house for the past 2 weeks?	☐☐
**Q11**	Household members	How many are in the following age groups	0-10 years ☐☐<br>11-19 years ☐☐<br>20-50 years ☐☐<br>50 years+ ☐☐
**Knowledge**			
**Q12**	CURE	There currently is no effective cure for COVID-19	True ☐<br>False ☐<br>I don't know ☐
**Q13**	SEVDIS	Not all persons with SARS-CoV-2 infection will develop to severe disease	True ☐<br>False ☐<br>I don't know ☐
**Q14**	RISK	Those who are elderly, have chronic illnesses, and are obese are more likely to get severe disease if they are infected with SARS-CoV-2	True ☐<br>False ☐<br>I don't know ☐
**Q15**	TRANS	Persons with SARS-CoV-2 infection cannot transmit the virus to others when symptoms are not present.	True ☐<br>False ☐

			I don't know ☐
**Q14**	ISOLATION	Isolation of people who are infected with SARS-CoV-2 is an effective way to reduce the spread of the virus.	True ☐<br>False ☐<br>I don't know ☐
**Q15**	QUARANTINE	People who have been in contact with someone infected with SARS-CoV-2 should be immediately isolated in a proper place. In general, the quarantine period is 14 days.	True ☐<br>False ☐<br>I don't know ☐
**Q15**	SOAP	Hand washing with soap for at least 40-60 sec is effective against SARS-CoV-2 infection	True ☐<br>False ☐<br>I don't know ☐
**Q16**	DECOM1	0.5% Sodium hypochlorite liquid (bleach) (5000 parts per million) can be used for decontamination of surfaces to inactive SARS-CoV-2	True ☐<br>False ☐<br>I don't know ☐
**Q17**	DECOM2	50% Ethanol can be used for decontamination of surfaces to inactive SARS-CoV-2	True ☐<br>False ☐<br>I don't know ☐
**Q18**	DECOM3	70% Ethanol can be used for decontamination of surfaces to inactive SARS-CoV-2	True ☐<br>False ☐<br>I don't know ☐
**Q19**	DECOM4	Boling water can be used for decontamination of surfaces to inactive SARS-CoV-2	True ☐<br>False ☐<br>I don't know ☐
**Q20**	Symptom	Which of the following are symptoms of COVID19?	Fever ☐<br>Headache ☐

			Cough ☐<br>Runny nose/congestion ☐<br>Fatigue ☐<br>Difficulty breathing ☐<br>Diarrhoea ☐<br>Loss of appetite ☐<br>Sneezing ☐<br>Joint pain ☐<br>Muscle pain ☐
**Q21**	DANGER	Let us say "COVID-19" refers to a "dangerous transmissible virus". Based on this meaning and your own observation, how serious do you think COVID-19 is at this moment.	SCALE 1-10
**Q22**	Feeling	How do you feel about Coronavirus?	Very fearful ☐<br>Fearful ☐<br>Fearful , but optimistic ☐<br>Neutral ☐
**Q23**		What is preventing you from full protecting yourself from SARS-CoV-2	
**Q23a**	Short_soap	Shortage of soap	Yes ☐<br>No ☐<br>Don't want to answer ☐
**Q23b**	Short_water	Shortage of water	Yes ☐<br>No ☐

			Don't want to answer ☐
**Q23c**	Short_ds	Shortage of hand sanitizer	Yes ☐<br>No ☐<br>Don't want to answer ☐
**Q23d**	Short_m	Shortage of masks	Yes ☐<br>No ☐<br>Don't want to answer ☐
**Q23e**	Short_g	Shortage of gloves	Yes ☐<br>No ☐<br>Don't want to answer ☐
**Q23f**	Price	Increasing of the price of items in the market	Yes ☐<br>No ☐<br>Don't want to answer ☐
**Q23g**	Wage	Loss or decrease of wages	Yes ☐<br>No ☐<br>Don't want to answer ☐
**Q23h**	Information	- Insufficient information	Yes ☐<br>No ☐<br>Don't want to answer ☐

Zhong B, Luo W, Li H, Zhang Q, Liu X, Li W, et al. Knowledge, attitudes and practices towards COVID-19 among Chinese residents during the rapid rise period of the COVID-19 outbreak: a quick online cross-sectional survey. Int J Biol Sci. 2020;16:1745–1752. pmid:32226294

Knowledge, Attitude and Practice Survey (Round 2) on COVID-19 response - UNICEF

**GX01**	*ID*	Study ID	IC□□□□□
**GX02**	*COLID*	Specimen collected by	□□
**GX03**	*DATE*	Date specimen collected (dd/MMM/yyyy)	□□/□□□/20□□
**GX04**	*TIME1*	Time of sputum collection (24hr clock)	□□:□□HRS

(FOR LAB USE ONLY below this line)

**GX05**	*DATER*	Date specimen was received (dd/MMM/yyyy)	□□/□□□/20□□
**GX06**	*LCODE*	Lab number	**Insert barcode**

**GX07**	*VOL*	Volume	☐mls
**GX08**	*QUAL*	Quality<br>**Tick all that apply**	Salivary☐ Mucoid☐ Mucopurulent☐ Blood Stained☐
**GX09**	*DATE1*	Date processed (dd/MMM/yyyy)	☐☐/☐☐☐/20☐☐
**GX10**	*PROCID*	Specimen processed by	☐☐
**GX11**	*MTB1*	MTB Detected	Yes ☐ No ☐ Indeterminate☐ Error ☐
**GX12**	*RIFR1*	Rifampicin sensitivity result	No MTB detected ☐<br>Sensitive ☐<br>Provisionally Resistant (must repeat to confirm) ☐<br>Rif result indeterminate ☐
**If ”Error” in GX11, or “Resistant” in GX12, repeat Xpert test by rerunning the <u>same sample</u> to confirm**			

**GX13**	*MTB2*	MTB detected on repeat test?	Yes ☐ No ☐ Indeterminate☐ Error ☐<br>Insufficient sputum to repeat test ☐
**GX14**	*RIFR2*	Rifampicin result from repeat test?	Sensitive ☐<br>Resistant ☐<br>Rif sensitivity result indeterminate ☐<br>Insufficient sputum to repeat test ☐
**GX15**	*MTBFIN*	Final MTB detected?	Yes ☐ No ☐ Indeterminate☐ Error ☐
**GX16**	*FINALR*	Final reported Rifampicin resistance	**Sensitive** (Sensitive on *EITHER* GX12 *or* GX14) ☐<br>**Confirmed resistance** (resistant on *BOTH* GX12*AND* GX14) ☐<br>**Provisional resistance** (resistant on GX12, indeterminate/insufficient sputum on GX14) ☐<br>**Not reported** (No MTB detected / Rif result indeterminate on GX12) ☐

....

Tear off

TB LABORATORY GENEXPERT RESULT: ICAROZ STUDY

Study ID: IC☐☐☐☐☐

Date of collection: ____________________ **Date Processed**_____________________________

Laboratory Number__________________

GeneXpert RESULT (to be completed in laboratory)

Visual appearance	Result (tick what applies)		
	Negative	MTB detected	Rifampicin resistance

Comments

____

____

ICAROZCRF Sars-COV2 Testing **ICAROZ-R04**

**S01**	*ID*	Study ID	IC☐☐☐☐☐
**S02**	*COLID*	Specimen collected by	☐☐
**S03**	*DATE*	Date specimen collected (dd/MMM/yyyy)	☐☐/☐☐☐/20☐☐
**S04**	*TIME1*	Time of specimen collection (24hr clock)	☐☐:☐☐HRS
**S05**	*Age*	Age	☐☐
**S06**	*Sex*	Sex	Male ☐ Female ☐
**S07**	*CONS*	Consent for Specimen Storage given (*Affix sticker if Yes*)	Yes ☐ No ☐

(FOR LAB USE ONLY below this line)

....

**S08**	*DATER*	Date specimen was received (dd/MMM/yyyy)	☐☐/☐☐☐/20☐☐
**S09**	*Lab*	Laboratory	AIBST ☐ BRTI ☐ LANCET ☐ Other ☐ Specify__________
**S10**	*LCODE*	Lab number	**Insert barcode or Write number**

**S11**	*DATE1*	Date processed (dd/MMM/yyyy)	☐☐/☐☐☐/20☐☐
**S12**	*EXTRACT ID*	Specimen extracted by	☐☐
**S13**	*AMPLYID*	Specimen amplified by	☐☐
**S14**	*COV*	SARS COV2 Detected	Yes ☐ No ☐ Invalid ☐ Error ☐
**S15**	*INFA*	Infuenza A Detected	Yes ☐ No ☐ Invalid ☐ Error ☐ NA ☐
**S16**	*INFB*	Infuenza B Detected	Yes ☐ No ☐ Invalid ☐ Error ☐ NA ☐
**S17**	*RSV*	RSV Detected	Yes ☐ No ☐ Invalid ☐ Error ☐ NA ☐
**S18**	*COCS*	Residual specimen stored	Yes ☐ No ☐ Insufficient specimen ☐
**S19**	*DATE2*	Date result complete (dd/MMM/yyyy)	☐☐/☐☐☐/20☐☐
**S20**	*STOR*	Speciment storage locations	Freezer No ☐☐☐ Box No ☐☐☐ Position ☐☐☐<br>Freezer No ☐☐☐ Box No ☐☐☐ Position ☐☐☐

Result Sheet

SARS-COV2-RESULTS: ICAROZ STUDY

Study ID: IC☐☐☐☐☐ **Laboratory Number**___________________

First Name:________________________ **Last Name:**________________________

Date of collection: ____________________ **Date Processed**_____________________________

SARS-COV2 RESULT (to be completed in laboratory)

Negative	Positive	Invalid	Other

Comments

Influenza A RESULT (to be completed in laboratory)

Negative	Positive	Invalid	Not done	Other

Comments

Influenza B RESULT (to be completed in laboratory)

Negative	Positive	Invalid	Not done	Other

Comments

RSV RESULT (to be completed in laboratory)

Negative	Positive	Invalid	Not done	Other

Comments

ICAROZ-FOLLOW-UP FORM **ICAROZ-R05**

**P0a**	ID	ID	IC☐☐☐☐☐
**P0b**	*COV*	SARS COV2 Detected	Yes☐No☐<br>Invalid☐Error ☐
**P0c**	*INFA*	Infuenza A Detected	Yes☐No☐Invalid☐Error ☐ NA ☐
**P0d**	*INFB*	Infuenza B Detected	Yes☐No☐Invalid☐Error ☐ NA ☐
**P0e**	*RSV*	RSV Detected	Yes☐No☐Invalid☐Error ☐ NA ☐
**Complete P01a – P01b for all clients**			
**P01**	DATE – 1st	Date contacted (1st attempt) (dd/mm/yyyy)	☐☐/☐☐☐/20☐☐
**P01a**	Time – 1st	Time contacted (1st attempt)	☐☐.☐☐
**P01b**	Success – 1st	Successful 1st attempt	Yes☐<br>No☐<br>SMS (for SARS-COV-2 negative)☐<br>Left message☐<br>Number not working☐
**Complete P02 – P05b for clients with any positive test results (SARS-CoV-2, RSV, Influenza A/B)**			
**P02**	DATE – 2nd	Date contacted (2nd attempt) (dd/mm/yyyy)	☐☐/☐☐☐/20☐☐
**P02a**	Time – 2nd	Time contacted (2nd attempt)	☐☐.☐☐
**P02b**	Success – 2nd	Successful 2nd attempt	Yes☐<br>No☐<br>Left message☐<br>Number not working☐

ICAROZ-FOLLOW-UP FORM **ICAROZ-R05**

**P03**	DATE – 3rd	Date contacted (3rd attempt) (dd/mm/yyyy)	☐☐/☐☐☐/20☐☐
**P03a**	Time – 3rd	Time contacted (3rd attempt)	☐☐.☐☐
**P03b**	Success – 3rd	Successful 3rd attempt	Yes☐<br>No☐<br>Left message☐<br>Number not working☐
**P04**	SMS/Whatsapp	Did you send SMS/Whatsapp to call back	Yes☐<br>No☐<br>Number not working☐
**P05**	Call back	Did the client call you back	Yes☐<br>No☐<br>Never called back ☐
**P05a**	DATE – call back	Date called back (dd/mm/yyyy)	☐☐/☐☐☐/20☐☐
**P05b**	Time – called back	Time called back	☐☐.☐☐
**P05c**	Min	Duration of discussion (min) – for those successfully contacted	☐☐
**Clinical – complete P06-P36 for clients testing SARS-CoV-2 positive only**			
**P06**	Fever	Do you have fever	Yes☐No ☐
**P07**	SOB	Do you have shortness of breath	On exertion☐<br>At rest☐<br>None☐
**P08**	Progress	How do you feel compared to when we saw you?	Completely resolved☐<br>Same☐<br>Better☐

			Worse☐<br>Don't want to answer☐		
**P09**	Symptom duration	How long have you had symptoms	________ DAYS		
**P10**	Counselling	Do you want us to put you in contact with counselling services	Yes☐No☐		
**P10b**	HHC	Is this a Household contact	Yes☐No ☐<br>**(if yes skip questions about HH members)**		
**HH members**					
**P11**	Adults	How many adults have been living with you since 2 days before your symptoms started?	☐☐		
**P12**	Children	Number of children (all children – not restricted to biological children) *(0 to 18 years)*	☐☐		
**P13**	Child 1	Child 1	Age☐☐	Female☐<br>Males☐<br>Don't want to answer☐	Symptoms<br>Yes☐<br>No☐<br>Don't want to answer☐
**P14**	Child 2	Child 2	Age☐☐	Female☐<br>Males☐<br>Don't want to answer☐	Symptoms<br>Yes☐<br>No☐<br>Don't want to answer☐
**P15**	Child 3	Child 3	Age☐☐	Female☐<br>Males☐<br>Don't want to answer☐	Symptoms<br>Yes☐<br>No☐<br>Don't want to answer☐
**P16**	Child 4	Child 4	Age☐☐	Female☐<br>Males☐<br>Don't want to answer☐	Symptoms<br>Yes☐<br>No☐<br>Don't want to answer☐
**P17**	Child 5	Child 5	Age☐☐	Female☐<br>Males☐<br>Don't	Symptoms<br>Yes☐<br>No☐

				want to answer☐	Don't want to answer☐
**P18**	Child 6	Child 6	Age☐☐	Female☐<br>Males☐<br>Don't want to answer☐	Symptoms<br>Yes☐<br>No☐<br>Don't want to answer☐
**P19**	Child 7	Child 7	Age☐☐	Female☐<br>Males☐<br>Don't want to answer☐	Symptoms<br>Yes☐<br>No☐<br>Don't want to answer☐
**P20**	Child 8	Child 8	Age☐☐	Female☐<br>Males☐<br>Don't want to answer☐	Symptoms<br>Yes☐<br>No☐<br>Don't want to answer☐
**P21**	Adult 1 rel	Adult 1 relationship	Spouse☐<br>Boyfriend/girlfriend☐<br>Parent☐<br>Sibling ☐<br>Domestic worker☐<br>Other relatives☐<br>Other non-relative☐<br>Specify ____		
**P21a**	Adult 1 info	Adult 1	Age☐☐	Female☐<br>Males☐<br>Don't want to answer☐	Symptoms<br>Yes☐<br>No☐<br>Don't want to answer☐
**P22**	Adult 2 rel	Adult 2 relationship	Spouse☐<br>Boyfriend/girlfriend☐<br>Parent☐<br>Sibling ☐<br>Domestic worker☐<br>Other relatives☐<br>Other non-relative☐		

			Specify ___		
**P22a**	Adult 2 info	Adult 2	Age☐☐	Female☐<br>Males☐<br>Don't want to answer☐	Symptoms<br>Yes☐<br>No☐<br>Don't want to answer☐
**P23**	Adult 3 rel	Adult 3 relationship	Spouse☐<br>Boyfriend/girlfriend☐<br>Parent☐<br>Sibling ☐<br>Domestic worker☐<br>Other relatives☐<br>Other non-relative☐<br>Specify ___		
**P23a**	Adult 3 info	Adult 3	Age☐☐	Female☐<br>Males☐<br>Don't want to answer☐	Symptoms<br>Yes☐<br>No☐<br>Don't want to answer☐
**P24**	Adult 4 rel	Adult 4 relationship	Spouse☐<br>Boyfriend/girlfriend☐<br>Parent☐<br>Sibling ☐<br>Domestic worker☐<br>Other relatives☐<br>Other non-relative☐<br>Specify ___		
**P24a**	Adult 4 info	Adult 4	Age☐☐	Female☐<br>Males☐<br>Don't want to answer☐	Symptoms<br>Yes☐<br>No☐<br>Don't want to answer☐
**P25**	Adult 5 rel	Adult 5 relationship	Spouse☐<br>Boyfriend/girlfriend☐<br>Parent☐<br>Sibling ☐		

			Domestic worker☐<br>Other relatives☐<br>Other non-relative☐<br>Specify ___		
**P25a**	Adult 5 info	Adult 5	Age☐☐	Female☐<br>Males☐<br>Don't want to answer☐	Symptoms<br>Yes☐<br>No☐<br>Don't want to answer☐
**P26**	Adult 6 rel	Adult 6 relationship	Spouse☐<br>Boyfriend/girlfriend☐<br>Parent☐<br>Sibling ☐<br>Domestic worker☐<br>Other relatives☐<br>Other non-relative☐<br>Specify ___		
**P26a**	Adult 6 info	Adult 6	Age☐☐	Female☐<br>Males☐<br>Don't want to answer☐	Symptoms<br>Yes☐<br>No☐<br>Don't want to answer☐
**P27**	Adult 7 rel	Adult 7 relationship	Spouse☐<br>Boyfriend/girlfriend☐<br>Parent☐<br>Sibling ☐<br>Domestic worker☐<br>Other relatives☐<br>Other non-relative☐<br>Specify ___		
**P27a**	Adult 7 info	Adult 7	Age☐☐	Female☐<br>Males☐<br>Don't want to answer☐	Symptoms<br>Yes☐<br>No☐<br>Don't want to answer☐

**P28**	Adult 8 rel	Adult8 relationship	Spouse☐<br>Boyfriend/girlfriend☐<br>Parent☐<br>Sibling ☐<br>Domestic worker☐<br>Other relatives☐<br>Other non-relative☐<br>Specify ___
**P28a**	Adult 8 info	Adult 8	Age☐☐ | Female☐ Males☐ Don't want to answer☐ | Symptoms Yes☐ No☐ Don't want to answer☐
**P29**	Vulnerable 1	Does anybody have diabetes as far as you know? Specify as adult 1 or 2	Yes ☐No☐Don't know☐<br>Specify (adult 1, adult 2)________
**P30**	Vulnerable 2	Does anybody have hypertension as far as you know?	Yes ☐No☐Don't know☐<br>Specify (adult 1, adult 2)________
**P31**	Vulnerable3	Does anybody have chronic kidney disease (dialysis) as far as you know?	Yes ☐No☐Don't know☐<br>Specify (adult 1, adult 2)________
**P32**	Symptomatic	Do any of the household members have symptoms suggestive of SARS-CoV-2?	Yes ☐No☐Don't know☐<br>NA☐
**P33**	Attend1	Is/are the symptomatic household member(s) able to attend the ICAROZ service?	Yes ☐No☐Don't know☐<br>NA☐
**P34**	Attend2	If the symptomatic household member(s) is unable to attend the ICAROZ services, do they want to be followed by a home visit?	Yes ☐<br>No☐
**Onward referral and follow-up**			
**P34b**	ICP pack	Does the case want/need an ICP pack?	Yes ☐

			No☐
**P35**	Referral	Does the index case need referral for COVID-19 now?	Yes ☐<br>No☐
**P36**	Follow-up	Does the index case need to be followed	Yes day 3 only ☐<br>Yes day 3 and day 7 ☐<br>No☐
**P37**	Date FU3	Date called for day 3 follow-up (dd/mmm/yyyy)	☐☐/☐☐☐/20☐☐
**P37a**	Success – FU3	Successful FU3 attempt	Yes☐<br>No☐<br>Left message (sms/ whatsapp)☐<br>Number not working☐
**P37b**	FU3_progress	How do you feel compared to when we spoke last?	Completely resolved☐<br>Same☐<br>Better☐<br>Worse☐<br>Don't want to answer☐
**P37c**	FU3_Referral	Does the index case need referral for clinical care for COVID-19 now?	Yes ☐<br>No☐
**P38**	Follow-up	Does the index case need/want to be followed at day 7?	Yes ☐<br>No☐
**P39**	Date FU7	Date called for day 7 follow-up (dd/mmm/yyyy)	☐☐/☐☐☐/20☐☐
**P39a**	Success – FU7	Successful FU7 attempt	Yes☐

			No ☐<br>Left message (sms/ whatsapp) ☐<br>Number not working ☐
**P39b**	FU7_progress	How do you feel compared to when we spoke last?	Completely resolved ☐<br>Same ☐<br>Better ☐<br>Worse ☐<br>Don't want to answer ☐
**P39 c**	FU7_Referral	Does the index case need referral for clinical care for COVID-19 now?	Yes ☐<br>No ☐
