# Supplementary material for: Comprehensive occupational health services for healthcare workers in Zimbabwe during the SARS-CoV-2 pandemic
Source: PLoS One. 2021 Nov 23;16(11):e0260261. doi: 10.1371/journal.pone.0260261 (PMC8610265; doi:10.1371/journal.pone.0260261)
Supplement: Attachment — Submitted filename: ICAROZ_Response to reviewers_2.docx [file pone.0260261.s005.docx]

Dear Editor,

Thank you for considering our manuscript for publication and for sending comments from peer reviewers. Please find below a detailed response to the editorial comments and reviewers’ comments. Please note that all line references refer to the “tracked changes” document attached (rather than the clean, unmarked PDF also provided).

1. Please ensure that your manuscript meets PLOS ONE's style requirements, including those for file naming. The PLOS ONE style templates can be found at <https://journals.plos.org/plosone/s/file?id=wjVg/PLOSOne_formatting_sample_main_body.pdf> and <https://journals.plos.org/plosone/s/file?id=ba62/PLOSOne_formatting_sample_title_authors_affiliations.pdf>

*We have double checked the requirements and have made the necessary changes.*

2. We note that you have indicated that data from this study are available upon request. PLOS only allows data to be available upon request if there are legal or ethical restrictions on sharing data publicly. For information on unacceptable data access restrictions, please see <http://journals.plos.org/plosone/s/data-availability#loc-unacceptable-data-access-restrictions>.

In your revised cover letter, please address the following prompts:

a) If there are ethical or legal restrictions on sharing a de-identified data set, please explain them in detail (e.g., data contain potentially identifying or sensitive patient information) and who has imposed them (e.g., an ethics committee). Please also provide contact information for a data access committee, ethics committee, or other institutional body to which data requests may be sent.

*We are committed to providing open access data, within the confines of producing a de-identified minimal dataset. All participants in the study are healthcare workers within a clearly identifiable (and named) healthcare facility, and there is a substantial risk that individuals could be identified because of a combination of demographic characteristics (e.g. sex and occupation). The principal investigator and collaborators felt that there was an unacceptable risk to participant confidentiality, particularly as these data include confidential health information (including about HIV status and other chronic illnesses). We have therefore produced a deidentified dataset in which information on occupation and sex has been removed, and ages replaced with age categories, permitting the reproduction of almost all analyses without compromising confidentiality*

b) If there are no restrictions, please upload the minimal anonymized data set necessary to replicate your study findings as either Supporting Information files or to a stable, public repository and provide us with the relevant URLs, DOIs, or accession numbers. Please see <http://www.bmj.com/content/340/bmj.c181.long> for guidelines on how to de-identify and prepare clinical data for publication. For a list of acceptable repositories, please see <http://journals.plos.org/plosone/s/data-availability#loc-recommended-repositories>.

We will update your Data Availability statement on your behalf to reflect the information you provide

*Please see above.*

3. Please provide additional details regarding participant consent. In the ethics statement in the Methods and online submission information please specify how verbal/oral consent was recorded. If your study included minors, please state whether you obtained consent from parents or guardians in these cases. If the need for consent was waived by the ethics committee, please include this information.

Furthermore, please include a copy of the baseline questionnaire used as a part of the study as Supporting File.

*Thank you. All participants were adults aged over 18; this has been added to the methods (line 116). Verbal consent was recorded electronically as part of the clinical questionnaire, with the ethics committees waiving a requirement for written consent; this is documented in the methods section (225-228).*

Major comments
1) The authors mention that the voluntary participation in the screening could introduce selection bias, which certainly exist. To that an important additional information is the size of the source/target population, which is not reported. How big is the source population, i.e. healthcare staff in Harare and at the Parirenyatwa Group of Hospitals?

*Parirenyatwa Group of Hospitals employs an excess of 2000 staff. However, most of the domestic workers and security staff are employed by private companies. In addition, students including those studying nursing, medicine and laboratory sciences, who had access to the screening service are not included in the staff estimates. Furthermore, while Parirenyatwa Group of Hospitals employs more than 2000 staff, the staff contingent actually working during the study period was much smaller. As outlined in the manuscript “As a result of the national lockdown, the facility moved to “emergency operations”, with outpatient services closed from the end of March 2020. Provision of other health services was limited by the industrial action by doctors and nurses.” Given the limitations regarding the size of the catchment population we are unable to provide an estimate about the proportion reached with this service. We have added a line to the methods to explain this (lines 105-106.)*

2) How was the occupational screening service advised? Did the advertisement reach the target population evenly?

*We have added a line in the methods to explain how the service was advertised (lines 127-128). All care was taken to ensure that news of the service reached all work groups, but this was a pragmatic study and data were not collected on how advertisement reached different groups.*

3) The title names front-line HCWs as the study population; however, the screening was offered free-of-charge for any HCWs, if I understand correctly. How was the participation of front-line HCWs assured?

*We appreciate that the distinction of a “front-line” healthcare worker may be misleading, so have removed this descriptor from the title and abstract (line 93). The service was set up to be inclusive from the start, i.e. we encouraged domestics, security staff and also people working as part of the estates (for example those working in the laundry and operating the incinerator) to access the services. These staff members may not be considered “front-line” health care workers, but may have a considerable risk of being exposed to SARS-CoV-2.*

4) “All clients were asked about symptoms of COVID-19 including fever, cough, coryzal symptoms, joint pain, headache and loss of smell or taste.” (page6, lines 19-20). How was the set of symptoms determined? E.g. fatigue and chest tightness are also typical symptoms of COVID-19 disease, but not on the list.

*Thank you – a fuller list of symptoms was also included but is not listed in full in the manuscript – please see questionnaire in Supplementary Materials for more information. At the time of recruitment there was not an internationally-accepted list of symptoms suggestive of possible COVID, so this list was derived from available guidance and consensus medical opinion.*

5) Who stayed out of SARS-CoV-2 testing? Were they different in any characteristics from those, who accepted testing?

*Few participants declined SARS-CoV-2 testing when offered (particularly as there was very limited availability of SARS-CoV-2 testing in Zimbabwe at the time), but data on this were not specifically collected. Of the 66 clients with COVID-19 symptoms, six were not tested. We have included the descriptive characteristics of those not tested for SARS-CoV-2, those testing negative, and those testing positive, as a supplementary table (Supplementary Table 1).*

6) “A further 23 clients without symptoms were also tested, of whom 20 (87%) reported contact with someone with COVID-19.” (page10, lines 18-19). It would be interesting to see how the proportion of positive PCR test results varied between groups of different indications for testing.

*We have included the descriptive characteristics (including contact history) of those not tested for SARS-CoV-2, those testing negative, and those testing positive, as a supplementary table (Supplementary Table 1).*

7) On Figure 1, the proportion of positive results from those tested would give a more valid picture since the absolute number of attendances to the screening service changed with time.

*Thank you for the suggestion. We wanted also to demonstrate the finding that, during the first weeks of the service, symptomatic clients predominated leading to a high proportion being tested, and that the balance of service users changed over time as well as the proportion testing positive, and therefore chose to present the data as in Figure 1.*

8) Similarly to the tested non-communicable disease, the potential risk factors of COVID-19 positivity could also be analysed (i.e. availability and use of preventive measures, knowledge and attitude), even though there were only a limited number of cases.

*We considered this, but the low number of cases precluded any meaningful statistical analysis and we therefore did not include this within the main body of the analysis. As the occupational health service continues to operate and to identify additional cases, this may be possible in future analyses. We have included the descriptive characteristics of those not tested for SARS-CoV-2, those testing negative, and those testing positive, as a supplementary table (Supplementary Table 1).*

9) Was there a question on the possible occupational exposure to COVID-19 patents in a healthcare setting? The authors speculate that most of the SARS-CoV-2 infections are probably community transmissions. It would be interesting to estimate it from available data.

*We agree that this is an interesting question. Our questionnaire only asked about contact to those with COVID-19, and did not differentiate between occupational and non-occupational exposure. This may be changed for future iterations.*

10) “All infections were non-severe, and many were pauci- or asymptomatic.” (page 17, lines 22-23). The study provides only a snapshot about the status of the positively tested participants. Is there follow-up information about the pauci- and asymptomatic cases? Some of them may have developed more severe symptoms later or became long-COVID cases.

*All participants were followed up until symptoms had fully resolved, and until 14 days from their positive test. We have expanded in the results (lines 264-5). Of course it is possible that some participants may have subsequently developed post-COVID syndromes, but at the time follow-up ended all participants were well.*

Minor comments
1) “While the clients waited for screening, a trained researcher administered a baseline questionnaire, which included questions on past medical history, knowledge about COVID-19 prevention and management, attitudes and behaviours related to COVID-19 and availability of PPE and other resources in their workplace.” (page 8, lines 2-5). What about the SSQ questionnaire? Was it not administered with the baseline questionnaire?

*Thank you – we have clarified in the methods section. Clients were asked to fill the SSQ questionnaire while they were waiting. The questionnaire was self-administered to ensure that clients had enough time to consider the questions and minimise social-desirability bias. The SSQ questionnaire includes questions which some people may not feel comfortable to answer truthfully when asked by another person, for examples questions on self-harm and crying. The client submitted to questionnaire to the nurse – who checked the score and looked for any answers which would raise red flags. We have clarified (lines 150-152).*

2) “Overall 7% (69/951) of clients reported at least one TB symptom but most were unable to produce a sputum sample.” (page 13, lines 16-17). What does “unable to produce a sputum sample” mean?

*Even amongst symptomatic, hospitalised or immunosuppressed cohorts with presumptive TB, a high proportion are unable to spontaneously expectorate a sputum sample. One might expect this proportion to be even higher amongst minimally-symptomatic clients identified in untargeted screening. For infection prevention and control purposes a decision was taken not to perform sputum induction.*

3) “Secondly, recruitment coincided…” (page 17, line 9). It is actually the third limitation.

*Thank you; amended (line 437).*

4) “We observed significant levels of SARS-CoV-2 infection amongst Zimbabwean healthcare workers,…” (page 17, lines 21-22). The significance of the prevalence of infection depends on the base for comparison. The rate is not high compared to many other places and times.

*We have edited this to “important” to avoid the suggestion of a statistical comparison (line 450).*
